# Avatar Embodiment. Towards a Standardized Questionnaire

**DOI:** 10.3389/frobt.2018.00074

**Published:** 2018-06-22

**Authors:** Mar Gonzalez-Franco, Tabitha C. Peck

**Affiliations:** ^1^Microsoft Research, Redmond, WA, United States; ^2^Mathematics and Computer Science Department, Davidson College, Davidson, NC, United States

**Keywords:** avatars, virtual reality, embodiment, questionnaires, body ownership illusion

## Abstract

Inside virtual reality, users can embody avatars that are collocated from a first-person perspective. When doing so, participants have the feeling that the own body has been substituted by the self-avatar, and that the new body is the source of the sensations. Embodiment is complex as it includes not only body ownership over the avatar, but also agency, co-location, and external appearance. Despite the multiple variables that influence it, the illusion is quite robust, and it can be produced even if the self-avatar is of a different age, size, gender, or race from the participant's own body. Embodiment illusions are therefore the basis for many social VR experiences and a current active research area among the community. Researchers are interested both in the body manipulations that can be accepted, as well as studying how different self-avatars produce different attitudinal, social, perceptual, and behavioral effects. However, findings suggest that despite embodiment being strongly associated with the performance and reactions inside virtual reality, the extent to which the illusion is experienced varies between participants. In this paper, we review the questionnaires used in past experiments and propose a standardized embodiment questionnaire based on 25 questions that are prevalent in the literature. We encourage future virtual reality experiments that include first-person virtual avatars to administer this questionnaire in order to evaluate the degree of embodiment.

## Introduction

In the real world, we experience our self as being inside a body that moves according to our intentions. Our body provides important social cues when interacting with others, as well as information about our location, posture, and self-perception of the world. Commonly when entering a fully-Immersive virtual environment (IVE) we cannot see our own body because our real-world view is covered by an opaque screen. However, a virtual body can be used to represent us inside the IVE. Self-avatars^1^, among other things, have been shown to positively impact memory and cognitive processing inside virtual reality (VR) (Steed et al., 2016). In that regard, not having a body in an IVE has the potential to negatively affect social, perceptual, and behavioral human performance, generating disembodied phenomena related to the minimal selfhood (Murray and Sixsmith, 1999; Blanke and Metzinger, 2009).

To overcome these problems, VR experiences can render self-avatars. A self-avatar is a collocated avatar that can replicate the user's body posture and motions using body-tracking systems (Spanlang et al., 2014). This self-avatar is experienced from a first-person perspective and, within the VR, provides a substitute body for the participant. An embodiment illusion is experienced when the participant indeed feels the illusion that the co-located self-avatar has effectively replaced their body at a physical and functional level while immersed in the IVE. However, there are several aspects that can affect the elicitation of the embodiment illusion:

### Location of the body

The location of the avatar in relation to the own body affects embodiment illusions. Research has shown that using an avatar that is collocated with the participant and shown from a first-person perspective with a head-tracked updated view (visual sensorimotor correlations) is enough to produce an *embodiment illusion*, even without full-body motion tracking (Maselli and Slater, 2013). The illusion persists in some occasions if the participant is then displaced from the self-avatar after a stimulation period; in those scenarios there is an out-of-body effect (Bourdin et al., 2017). The avatar must be collocated with the user in order to experience an embodiment illusion.

### Body ownership

The user's perception of owning the avatar's body affects embodiment illusions. Body ownership can be induced in both collocated and non-collocated avatars. As described originally by the Rubber Hand Illusion (RHI) (Botvinick and Cohen, 1998), body ownership can be induced through multisensory integration. When visuo-tactile stimuli are provided synchronously to an artificial and own body part, participants perceive the artificial body part to be their own and that the tactile stimulation on the artificial body part is the source of their sensations. The working mechanism for the embodiment illusion includes the use of multisensory integration. For example visuo-tactile sensation can be used to elicit body ownership of a body part: users can start experiencing strong body ownership when a virtual object touches the virtual body at the same time and place as the user's real body (Slater et al., 2009). Users must experience ownership over the virtual body to experience an embodiment illusion.

### Agency and motor control

Embodiment illusions can be enhanced by providing users agency over the virtual body. Full-body visuo-motor synchronous stimulation may be used to induce further embodiment; i.e., having the virtual and real bodies move synchronously (Kokkinara and Slater, 2014). Indeed, movement of the body parts (more directly related to agency) has been shown to further enhance embodiment, perhaps with a stronger influence of the illusion than visuo-tactile stimulation alone since the former only tackles body ownership while the later addresses both agency and body ownership (Tsakiris et al., 2006; Kokkinara and Slater, 2014). The illusion can additionally be enhanced by placing virtual mirrors in front of the self-avatars (Gonzalez-Franco et al., 2010).

### External appearance

The appearance of the avatar may enhance or inhibit the embodiment illusion. Embodiment is not only elicited in look-alike or gender/race consistent self-avatars, but is also possible with avatars of a different gender (Kilteni et al., 2013), shape (Yee et al., 2009; Normand et al., 2011; Kilteni et al., 2012b; Won et al., 2015), racial group (Peck et al., 2013), or age (Banakou et al., 2013).

In summary, to experience an embodiment illusion, users must perceive the avatar as collocated with their own body, and that they own the body. The appearance and control of the avatar enhances the embodiment illusion.

Derived from these experiments, it is clear that the sense of embodiment is complex, and involves several aspects. It includes the recognition of an own perspective of the world, the experience of owning the body (body ownership), which can be influenced by the external appearance of the body and the ability to control the actions of the body (agency), and the possibility to feel the sensorial events directed to the body (such as touch) (Carruthers, 2008; Longo et al., 2008; Kilteni et al., 2012a).

Through the use of different techniques of stimulation, experimenters have shown it is possible to induce embodiment illusions in a wide variety of participants. However, as with other illusions, such as the RHI, the level of embodiment varies among participants and varied reactions and attitudinal changes to the embodiment experience is observed.

Research has shown that plasticity and attitudinal changes take place only if the embodiment illusion is enabled. For example, Caucasian participants reduced their racial bias after embodying black avatars (Peck et al., 2013). Embodying taller avatars increased user ability to negotiate more confidently, compared to users embodied in shorter avatars (Yee and Bailenson, 2007; Yee et al., 2009). Professionally-dressed self-avatars reduced participant's musicality when playing the bongo (Kilteni et al., 2013). Users even changed their saving behavior after embodying avatars that looked like older versions of themselves (Hershfield et al., 2011), and showed a modified psychological treatment behavior when embodying an avatar representing Sigmund Freud (Osimo et al., 2015). The use of self-avatars in VR impacts human behavior and therefore measuring the extent to which self-avatars are embodied is a critical aspect to the further exploration of VR experiences and their effects on future users.

## Toward a standardized embodiment questionnaire

Previous experiments have included both qualitative and quantitative metrics to measure the embodiment of avatars. Questionnaires, heart-rate monitors, skin-conductance, and electroencephalogram (EEG) are some of the techniques more commonly used. The physiometric measures support embodiment when people respond to actions performed on the virtual body in the same way as if the action was performed on the own body. For example, using EEG the same region in the brain that responds to threat was activated when an embodied virtual hand was stabbed with a virtual knife (González-Franco et al., 2014). Even though the evaluation of a perceptual illusion via a subjective questionnaire that is delivered after the fact may not render the best results (Slater, 2004), questionnaires are still the most prevalent metric, due to versatility and ease of use. Additionally, several embodiment experiments that use questionnaires as well as quantitative measures have shown correlations between the objective effects of the experiment and the subjective embodiment levels of the participants as extracted from the questionnaires (González-Franco et al., 2014; Padrao et al., 2016). Therefore, comprehensive questionnaires may render a reasonable embodiment measure.

However, despite the growing usage of self-avatar embodiment among the VR community, and the prevalence of questionnaires to measure perceived embodiment, there is not yet a standardized embodiment questionnaire.

One challenge in creating a standardized embodiment questionnaire arises from the different nature of the inducement of embodiment through visuo-tactile, visuo-motor, and other multisensory integration stimulation. Additionally, different hardware and lab setups makes standardized questionnaires a bit more challenging. However, the use of a standardized questionnaire will enable researchers to compare embodiment results over these different setups and enable a better understanding of what sensations are needed to most effectively induce an embodiment illusion or to replicate particular attitudinal responses.

Many current embodiment questionnaires are modified versions of the original RHI questionnaire, adapted to the design of the virtual experience to include, in addition to body-ownership, aspects such as agency. For example, some adaptations account for a whole-body substitution in which the body is collocated and in front of a virtual mirror (Slater et al., 2010b; Borland et al., 2013), compared to a dislocated body part shown in experiments with the RHI (IJsselsteijn et al., 2006; González-Franco et al., 2014). Yet, the many variations of the questionnaire in the literature make it impossible to compare results across experiments.

Hence, it is very important for the advancement of self-embodied avatars in VR to marshal a standardized embodiment questionnaire. Not only to evaluate the effects of one's own experiments, but also to be able to draw comparisons and replicate the experiences that are delivered by different experiments in a more standard way.

In this paper, we compile and classify questions that have been used in previous experiments to measure embodiment in different scenarios and recommend a standardized questionnaire for future use.

## Review

We first review over 30 embodiment experiments that have used questionnaires since 1998, when the RHI experiment was first published (Botvinick and Cohen, 1998) (see Appendix 1 in Supplementary Material). Work from different research laboratories that study body ownership illusions with and without VR around the world have been revisited in the process of this review. The review includes experimental questionnaires from the EVENTLab (The Experimental Virtual Environments Lab for Neuroscience and Technology) at University of Barcelona; the VECG (Virtual Environments and Computer Graphics) at University College London; LNCO (Laboratory of Cognitive Neuroscience) at EPFL (École POLYTECHNIQUE FÉDÉRALE DE LAUSANNE); The body and brain—Ehrsson Lab in Karolinska Institutet; Institut for Cybernetics at Max Plank; Virtual Human Lab in Stanford; the Computer Science department at the University North Carolina at Chapel Hill; the Virtual Embodiment lab in Cornell; and the French INRIA. There are certainly other groups exploring bodily illusions. Our lab inclusion criteria was based on the number and citation count of their publications in this topic area. To do a systematic search we started using a temporal criteria considering the seminal embodiment work on the RHI (Botvinick and Cohen, 1998). This was the first work suggesting the possibility of embodiment illusions and the first work proposing an embodiment type questionnaire. As we consider this to be the seminal work in the field, we narrowed our search by only considering papers that both referenced this seminal work and used virtual reality. Therefore, the review is restricted to only works from 1998 till 2017 that cited the original RHI and used virtual reality to induce embodiment.

As part of the exercise to come up with a standard questionnaire, we have classified all the questions in Appendix 1 in Supplementary Material. We have identified 6 main types of questions that are present depending on the experimental setup:

1. **Body ownership**. Present whenever there is a substitute body or body part. It is possible to have body ownership over a body that participants feel is not in the same location as their own body.
2. **Agency and motor control** of the body. Present whenever there is motion tracking and the participant can move parts or all of the virtual body.
3. **Tactile sensations**. Present whenever there is tactile or haptic stimulation to enhance the embodiment illusion.
4. **Location of the body**. Present whenever there is a substitute body or body part that is either collocated or not collocated with the participant. Participants must feel that their body is in the same location as the virtual body in order to experience an embodiment illusion. Participants may sense an out-of-body effect, or that the location of their body has drifted toward the location of the avatar. These questions are often only asked when the avatar is not collocated with the participant.
5. **External appearance**. Present when the self-avatar is a look-alike avatar or as control questions when there are shape, gender, race, clothing, or other visual modifications to the avatar different from the self.
6. **Response to external stimuli**. In many occasions during the experiment there is an event that modifies or threatens the body or body parts of the self-avatar. If the participant is embodied in the self-avatar then the participant will react as if their own body is threatened. This is often measured through both questionnaire and quantitative response.

This classification is also in agreement with previous research that has proposed this distinction of question types, including ownership, location, and agency (Kilteni et al., 2012a; Piryankova et al., 2014). Within the classification, some questions can be arranged in two or more types of categories; e.g. a question may be classified as both types 3 and 4 when a tactile sensation modifies the location of the body. Additionally, classification 1 about body ownership is dependent on the use of either collocated or not collocated avatars; since not collocated avatars can generate an Out-of-body illusion (Ehrsson, 2007; Lenggenhager et al., 2007; Bourdin et al., 2017). Co and not collocated avatars must be used independently.

When looking at these 6 categories we find that 96% of the analyzed studies (Appendix 1 in Supplementary Material) asked questions about **Body ownership**.

The increased use of **Agency and motor control** questions have over time aligned with the appearance of body-tracking systems (Spanlang et al., 2014). This trend shows that researchers are now using less visuo-tactile and haptic tricks to enhance the embodiment illusion and more visuo-motor stimulation. Studies support this trend and have shown that synchronous visuo-motor correlations provide stronger embodiment illusions than only visuo-tactile stimulation (Kokkinara and Slater, 2014). Moreover, participants unable to control the self-avatar cannot interact properly with the virtual world and therefore are unable to experience full embodiment (Kilteni et al., 2012a). Therefore, it is safe to assume that using visuo-motor stimulation to produce the embodiment illusion has other positive side effects, such as being able to control the virtual body.

**Tactile sensations** questions were used by 57% of the studies, specially by those prior to 2014, while the more recent studies, 49% of studies surveyed, asked questions about Agency of the body. In most cases these two sets of questions were exclusive as either one type of multisensory integration (either visuo-tactile or visuo-motor) was used to generate the illusion of embodiment. In particular, when participants could not move the self-avatar the agency questions were not relevant.

**Location of the body**. Additionally, 48% of the studies asked questions regarding the location of the body. In many cases, these questions are relevant when the body is not in the same location as the self-avatar, seen from a third person perspective, or when alterations in space perception are expected. We recommend that all embodiment questionnaires include a location of the body question, regardless of avatar proximity to the user, as collocation of the avatar is critical for an embodiment illusion.

Questions related to the external appearance of the avatar's body in comparison to the self as control questions are present in 60% of studies. These questions asked if the participant was “turning into” the virtual body or if the participant felt they were wearing the same clothes as the avatar. These questions are often thought of as control questions (i.e., no significant changes were expected), we believe that they will gain importance now that it is possible to further alter the appearance of self-avatars.

**Response to external stimuli** questions were present in 36% of the cases. They are generally linked to particular events in the experience, such as bodily threats or attacks. Despite being the least prevalent category on the reviewed experiments, behavioral responses are often measured in addition to using questionnaires. More importantly several experiments have shown correlations between questionnaires and physiological and behavioral responses (Maselli and Slater, 2013; González-Franco et al., 2014) thus supporting the relevance of questionnaires. Note that presenting response questions after each condition in within subject studies might reveal the aim of the experiment and influence the participant response to the next condition.

## Proposal of embodiment questionnaire

After revisiting the embodiment questionnaires that have been used in the last decades (Appendix 1 in Supplementary Material), we identify a set of questions to be standardized for future embodiment experiences, yet to be as backwards compatible as possible to enable comparison to previous experiments. We organize the questions as one of each of the 6 types of experimental interests that were previously identified (body ownership, agency and motor control, tactile sensations, location of the body, external appearance, and response to external stimuli).

When future experimenters use this questionnaire, they might choose a subset of questions with the same rationale. E.g., if an experiment does not involve agency or control of the body, those questions would not be needed. However, we recommend to administer the full 25-question and possibly not analyze some questions *a posteriori*, set so it is easier to draw comparisons across experiments.

In the following list we present a compilation of questions from Appendix 1 in Supplementary Material that address the different aspects that affect virtual embodiment:

1. Body ownership.
  Q1. “I felt as if the virtual ____was my ____”
  If there is more than one avatar, e.g. in a VR social interaction, use a longer version: “I felt as if the virtual ____I saw when I looked down was my ____”
  Q2. “It felt as if the virtual ____I saw was someone else”
  Q3. “It seemed as if I might have more than one ____”
  - If there is a mirror:
  Q4. “I felt as if the virtual ____I saw when looking in the mirror was my own ____”
  Q5. “I felt as if the virtual ____I saw when looking at myself in the mirror was another person”
2. Agency and motor control.
  Q6. “It felt like I could control the virtual ____as if it was my own ____”
  Q7. “The movements of the virtual ____were caused by my movements”
  Q8. “I felt as if the movements of the virtual ____were influencing my own movements”
  Q9. “I felt as if the virtual ____was moving by itself”
3. Tactile sensations.
  Q10. “It seemed as if I felt the touch of the ____ in the location where I saw the virtual ____touched”
  Q11. “It seemed as if the touch I felt was located somewhere between my physical ____and the virtual ____”
  Q12. “It seemed as if the touch I felt was caused by the ____ touching the virtual ____”
  Q13. “It seemed as if my ____ was touching the ____”
4. Location of the body.
  Q14. “I felt as if my ____was located where I saw the virtual ____”
  Q15. “I felt out of my body”
    - If the virtual body is not collocated with the participants' body:
  Q16. “I felt as if my (real) ____were drifting toward the virtual ____or as if the virtual ____were drifting toward my (real) ____”
5. External appearance.
  Q17. “It felt as if my (real) ____were turning into an ‘avatar’ ____”
  Q18. “At some point it felt as if my real ____was starting to take on the posture or shape of the virtual ____that I saw”
  Q19. “At some point it felt that the virtual ____resembled my own (real) ____, in terms of shape, skin tone or other visual features.”
  Q20. “I felt like I was wearing different clothes from when I came to the laboratory”
6. Response to external stimuli.
  Q21. “I felt that my own ____could be affected by ____”
  Q22. “I felt a ____sensation in my body when I saw ____”
  Q23. “When ____ happened, I felt the instinct to ____”
  Q24. “I felt as if my ____had ____”
    - If there is a threat to the body:
  Q25. “I had the feeling that I might be harmed by the ____”

^*^When using the questionnaire replace ____ with “body,” “arm” or an appropriate body representation.

Some subsets of questions might not be applicable to some experiments. And some experiments might still require additional questions. For example, if there is no tactile or haptic interaction the tactile questions might not be needed. In some cases, it may make sense to change the reference to the whole body in the questions for a specific body part or region of interest (hand, etc).

Ideally the experimental design will include these questions in a randomized order to limit context effects, and using a 7-point Likert-scale directly at the end of the experiment or of each condition if the study is within subjects. The Likert-scale should range from:

*strongly disagree (*−*3), disagree (*−*2), somewhat disagree (*−*1), neither agree nor disagree (0), somewhat agree (*+*1), agree (*+*2), strongly agree (*+*3)*

At the beginning of the questionnaire, it should be clear that the questions are related to the participants' experience during the experiment. Starting the questionnaire with a sentence of the style: “During the experiment there were moments in which…” could help (see Appendix 2 in Supplementary Material for the ready-to-print questionnaire).

If the results want to be presented as a single embodiment score, rather than on a per question basis, we propose that the values be aggregated using Principal Component Analysis (PCA), we provide a sample code on how to complete the embodiment PCA as part of this paper^2^ Alternatively if main effects are not to be taken into account, the users of this questionnaire could directly proceed with an arithmetic addition of scores as follows:

1. Ownership = (Q1 − Q2) − Q3 + (Q4 − Q5)
2. Agency = Q6 + Q7 + Q8 − Q9
3. Tactile sensations = (Q10 − Q11) + Q12 + Q13
4. Location = Q14 − Q15 + Q16
5. Appearance = Q17 + Q18 + Q19 + Q20
6. Response = Q21 + Q22 + Q23 + Q24 + Q25

Total Embodiment = ((Ownership/5) ^*^ 2 + (Agency/4) ^*^ 2 + Tactile Sensation/4 + (Location/3) ^*^2 + Appearance/4 + Response/5) / 9.

This formula emphasizes the key aspects of embodiment: Ownership, Agency, and Self-location by weighting those questions higher (Kilteni et al., 2012a; Piryankova et al., 2014).

If administered directly after each condition in the experimental setup the questionnaire should be directly comparable both within and between subjects and conditions. For the analysis and result presentation non-parametric tests should be used as these are not continuous variables. Examples of the non-parametric tests are also included in the supplementary sample code.

We also recommend some basic consistency checks to all experimenters: under normal circumstances Q2 should be scored as the inverse of Q1. Similarly, Q4 and Q5 should be paired with Q5 be scored as the inverse of Q4.

## Prevalence

In this section we study both the prevalence and experimental significance of the selected questions in previous experiments (Table 1 and Appendix 1 in Supplementary Material).

**Table 1:** Prevalence of the proposed questions in the literature (represented by Appendix 1 in Supplementary Material).

	**Question**	**Previously used in Appendix 1 in Supplementary Material**	**Prevalence**
Ownership	Q1	1Q3, 2Q1, 4Q3, 6Q3, 7Q3, 8Q1, 9Q1, 11Q3, 12Q3, 13Q1, 14Q3, 15Q3, 17Q3, 18Q8, 19Q1, 20Q3, 21Q2, 22Q1, 23Q1, 24Q1, 24Q2, 24Q14, 25Q1, 28Q2, 29Q1, 31Q1, 32Q6, 33Q1 Long version: 18Q2, 22Q1, 24Q3, 26Q1, 27Q1, 30Q1, 33Q5	100% of the experiments asked this question.
	Q2	5Q10, 12Q9, 17Q8, 19Q5, 21Q8, 23Q3, 25Q4, 28Q6, 32Q7, 33Q2	30%
	Q3	1Q5, 4Q5, 5Q7, 6Q5, 7Q8, 11Q5, 12Q5, 13Q10, 14Q7, 15Q4, 17Q5, 20Q5, 21Q7, 22Q4, 23Q4, 24Q9, 26Q4, 29Q3, 30Q2, 31Q5	66%
	Q4	9Q5, 10Q1, 18Q1, 18Q7, 22Q2, 26Q2, 26Q3, 27Q2, 30Q3	27% (it is a significant prevalence since not all experiments provided a mirror)
	Q5	10Q4	3% (it is a good consistency check for Q6)
	Q6	2Q2, 10Q3, 16Q1, 19Q4, 22Q7, 24Q26, 29Q9, 32Q5	24% (It is a significant since agency related questions are only used if there is motor control over the virtual body)
Agency	Q7	21Q3, 22Q5, 26Q5, 27Q3, 28Q5, 29Q5, 30Q5, 31Q2, 31Q4	27%
	Q8	29Q6, 22Q10	6% (this question is particularly interesting if there are motor alterations introduced to the virtual body)
	Q9	18Q3, 22Q9, 29Q7, 32Q8	12%
	Q10	1Q1, 4Q1, 6Q1, 7Q1, 11Q1, 12Q1, 13Q3, 14Q1, 15Q1, 17Q1, 20Q1, 23Q12, 24Q21, 31Q3	42%
Tactile sensations	Q11	1Q6, 5Q3, 7Q5, 4Q6, 11Q6, 14Q4, 15Q5, 17Q6, 20Q6, 24Q19	30%
	Q12	1Q2, 4Q2, 6Q2, 7Q2, 9Q3, 11Q2, 12Q2, 13Q2, 14Q2, 17Q2, 20Q2, 21Q4, 23Q11, 24Q22	42%
	Q13	5Q8, 9Q2, 15Q2, 24Q20, 31Q2	15%
	Q14	3Q1, 21Q1, 24Q24, 28Q1, 29Q2	15%
Location of the body	Q15	5Q5, 10Q5, 29Q4, 32Q4, 33Q7	15%
	Q16	1Q4, 1Q8, 4Q4, 4Q7, 7Q4, 7Q7, 11Q4, 11Q8, 17Q4, 17Q7, 20Q4, 20Q7, 24Q5	21%
	Q17	1Q7, 2Q1, 6Q6, 7Q6, 11Q7, 12Q6, 13Q6, 29Q8	24%
External appearance	Q18	14Q5, 14Q10, 15Q6, 23Q8, 24Q12, 28Q3	15%
	Q19	1Q9, 2Q2, 6Q7, 7Q8, 11Q9, 12Q7, 13Q7, 22Q3, 30Q4	27%
	Q20	5Q1, 6Q4, 9Q4, 12Q4, 13Q5, 23Q2	18%
	Q21	9Q8, 21Q6, 25Q5, 32Q10	12%
Response to external stimuli	Q22	19Q2, 21Q5, 23Q9, 32Q2	12%
	Q23	19Q6, 23Q10, 32Q11	9%
	Q24	13Q5, 14Q9, 24Q4, 28Q7	12%
	Q25	10Q2, 17Q9, 25Q2, 32Q9	12%

Some previously-used questions that had very low prevalence and or significance (see Appendix for significance details) were discarded in our proposed questionnaire. Such as “When I looked at the avatar I had a strong connection as if I was looking at myself” (9Q6, 11Q10). “I felt my own body had disappeared” (5Q2, 33Q6). “It felt as if I was at two places at the same time” (5Q6, 23Q7). “It felt that the virtual body was appropriate for the task” (18Q6, 18Q7). “I had a conflict between my body and the seen body” (14Q6, 24Q8). “I felt more comfortable/xxxx with my body than I normally am” (18Q4, 24Q10). “The virtual body I saw looked like me” (27Q4). “I felt that if something were to happen to the avatar it was like it was happening to me” (8Q2, 9Q7).

Other questions were grouped. For example, Q13 “It seemed as if my body was touching the virtual xxx,” it is meant to combine also the questions “I had the feeling that the xxx was directly touching me” (5Q8, 9Q2, 24Q20) and “It seemed as if the touch I felt was from the xxx being touched by the virtual body” (15Q2).

For the particular case of Q17, most of the experiments present this question split into two: “I felt as if my (real) body were drifting toward the virtual body” (1Q4, 4Q4, 7Q4, 11Q4, 17Q4, 20Q4, 24Q5). “I felt as if the virtual body were drifting toward my (real) body” (1Q8, 4Q7, 7Q7, 11Q8, 17Q7, 20Q7). However, in order to reduce the questionnaire, we propose to combine them into one. This way we ensure that if there is a drift it is accounted for. Nevertheless, if the particular interest of the embodiment study was to explore the drift directionality, then using two different questions would be appropriate.

## Embodiment, presence, and immersion

Several studies have shown that using embodied avatars can lead to increases of the subjective sense of presence inside VR, increasing both the place illusion and the plausibility of the experience (Slater, 2009; Slater et al., 2010a). The increase of presence illusion also translates into stronger immersion (Slater et al., 2010a; Skarbez et al., 2017).

From an objective perspective this makes sense particularly for social VR: having a body is an important aspect of social interaction, since it allows us to keep peripersonal spaces (proxemics) or show expressions when interacting with others (Llobera et al., 2010; Trutoiu et al., 2011; Kastanis and Slater, 2012; Li et al., 2015).

But more deeply having a body in VR also helps to create more natural interactions and can change our sense of space, affect our distance estimation, and even our cognitive load (Ries et al., 2008; Steed et al., 2016; Gonzalez-Franco and Lanier, 2017). At the end of the day in reality we do not exist without a body and being present somewhere is very much a bodily experience. It has been argued that when participants are disembodied they can generate illusory invisible body ownership illusions to compensate (Guterstam et al., 2013).

Therefore, it will not be surprising that experiments that deliver poor embodiment on avatars, possibly due to poor tracking setups will also reduce the presence illusion of participants and even reduce the plausibility illusion (Slater et al., 2010a; Spanlang et al., 2014).

## Discussion

The current paper proposes a standard questionnaire to measure embodiment to self-avatars in VR (see Appendix 2 in Supplementary Material for the ready to print questionnaire). It does so by compiling and reviewing the questionnaires of over 30 of the most relevant studies in the field over the two decades. It also considers the practices and schools of thought of different laboratories globally. Virtual embodiment is becoming a popular research field with many labs researching the effects and methods of embodiment, and many more papers could have been included in this survey. This review is restricted to only works from 1998 till 2017 that cited the seminal embodiment work on the RHI (Botvinick and Cohen, 1998) and used virtual reality to induce embodiment. Adding additional studies could potentially be used in the future to identify additional questions that might supplement this set.

In the process of standardization, we first identified six sets of variables that influence global aspects of embodiment: body ownership, agency and motor control, tactile sensations, location of the body, external appearance, and response to external stimuli. All of these aspects have been previously related to be affected by, or to be good measures of embodiment (Kilteni et al., 2012a; Maselli and Slater, 2013). In the review, we also present the percentage of use of these sets of questions in the literature. The prevalence of use helped us narrow down the number of questions to 25.

Although the current questionnaire has not been tested nor validated *per se*, it consists of questions that were prevalently used in prior studies and that produced significant results for experimenters to demonstrate the participants were embodied in avatars under specific conditions. In that sense this questionnaire is backwards compatible. The proposed questions are linked to particular experiments in Appendix 1 in Supplementary Material and in Table 1. Therefore, the validity of this questionnaire rests on the validity of previous research that has found these questions significant (check Appendix 1 in Supplementary Material to see all questions that were analyzed one by one for all publications: their scores and whether they were important descriptors). Further validation from the community would be desirable. We also acknowledge that additional questions might be needed for specific purposes.

In order to calculate the Embodiment Score, we propose a simple aggregation method. But we encourage others in the community to propose their own aggregation functions or to use PCA analysis, the sample code for the PCA analysis is provided along with this paper^2^.

We hope that this compilation is standard enough, and flexible enough to become a guide for future researchers. Hopefully, this will aid studying more the effects of particular VR experiences in participants that exhibit different embodiment levels.

This questionnaire is meant to be administered at the end of every study that involves an avatar to represent participants. It is important to emphasize how radically different the effects of the experience can be depending on the degrees of embodiment that participants experience. Aspects such as participant's behavior or their physiology are heavily influenced by the embodiment score (González-Franco et al., 2014; Padrao et al., 2016; Slater and Sanchez-Vives, 2016). The embodiment itself can be modulated by the type, race and look of the avatar participants embody (Hershfield et al., 2011; Kilteni et al., 2013; Peck et al., 2013). Therefore, potentially any manipulation to the participants' avatar might have strong changes on their performance during and after their VR experience.

## Author contributions

MG-F and TCP wrote and conceived the paper. Both contributed to the final questionnaire selection. MG-F completed the appendix review of previously used questions and provided the sample code for the PCA analysis of questionnaires.

### Conflict of interest statement

MG-F is an employee of Microsoft Research, the author declares that the current paper presents a balanced and unbiased review on the questionnaires used to measure embodiment of avatars, and that the review was conducted following scientific research standards. The remaining author declares that the research was conducted in the absence of any commercial or financial relationships that could be construed as a potential conflict of interest.

## Footnotes

^1^When we refer to a self-avatar, we mean any virtual or real representation of a body that someone may accept as an own body. This includes virtual bodies and mannequins in part or whole.

^2^https://notebooks.azure.com/margon/libraries/EmbodimentQuestionnairePCA
