# Supplementary material for: Avatar Embodiment. Towards a Standardized Questionnaire
Source: Front Robot AI. 2018 Jun 22;5:74. doi: 10.3389/frobt.2018.00074 (PMC7805666; doi:10.3389/frobt.2018.00074)
Supplement: Supplementary file 1 [file Data_Sheet_1.PDF]

Appendix 1:

Previous experiments and questions classified by types: (1) Body ownership. (2) Agency and motor control of the body. (3) Tactile sensations. (4) Location of the body. (5) External appearance. (6) Response to external stimuli.

Paper	Q1	Q2	Q3	Q4	Q5	Q6	Q7	Q8	Q9	Q10
Botvinick M and Cohen J (1998) “Rubber hands 'feel' touch that eyes see” Nature 391, 756<br><br>(Botvinick and Cohen, 1998)	(3)(4) It seemed as if I were feeling the touch of the paintbrush in the location where I saw the rubber hand touched<br><br>Score 3 (from -3 to 3) Significant difference from the middle score (0)*	(3) It seemed as if the touch I felt was caused by the paintbrush touching the rubber hand<br><br>Score 2.5<br>*	(1) I felt as if the rubber hand were my hand<br><br>Score 2.5<br>*	(4) I felt as if my (real) hand were drifting towards the right (towards the rubber hand)<br><br>Score -3<br>*	(1) It seemed as if I might have more than one left hand or arm<br><br>Score -2.5<br>*	(3)(4) It seemed as if the touch I was feeling came from somewhere between my own hand and the rubber hand<br><br>Score -1	(1)(5) It felt as if my (real) hand were turning 'rubbery'<br><br>Score 0	(4) It appeared (visually) as if the rubber hand were drifting towards the left (towards my hand)<br><br>Score -2.5<br>*	(5) The rubber hand began to resemble my own (real) hand, in terms of shape, skin tone, freckles or some other visual feature.<br><br>Score 0.5	
Lok, Benjamin, et al. (2003) "Effects of handling real objects and avatar fidelity on cognitive task performance in virtual environments." IEEE VR	(1)(5) How much did you associate with the visual representation of yourself (your avatar)?	(1) How realistic (visually, kinesthetically, interactivity) was the visual representation of yourself								

(Lok et al., 2003)	Score m=4.64, sd=1.65 (from 1 to 7) Significant difference from the middle score (4)*	(your avatar)?<br><br>Score m=4.5 sd=1.74								
IJsselsteijn, Wijnand A., et al. (2006) "Is this my hand I see before me? The rubber hand illusion in reality, virtual reality, and mixed reality." Presence 15.4: 455-464.<br><br>(IJsselsteijn et al., 2006)	(3)(4) It seemed as if I were feeling the touch in the location where I saw the rubber hand touched<br><br>Score 4.5 (from 1 to 7)	(3) It seemed as if the touch I felt was caused by the paintbrush touching the rubber hand<br><br>Score 5.5 Significant difference from the middle score (4)*	(1) I felt as if the rubber hand were my hand<br><br>Score 4.5	(4) I felt as if my hand were drifting towards the rubber hand<br><br>Score 2.5 *	(1) It seemed as if I had more than one left hand or arm<br><br>Score 2.5 *	(3)(4) It seemed as if the touch I was feeling came from somewhere between my own hand and the rubber hand<br><br>Score 2.5 *	(1)(5) It felt as if my hand were turning 'rubbery'<br><br>Score 1.5 *	(4) It appeared as if the rubber hand were drifting towards my hand<br><br>Score 1.5 *	(5) The rubber hand began to resemble my hand in form.<br><br>Score 4	(1) It felt as if my hand were in the rubber hand.<br><br>Score 3
Lenggenhager, B., et al (2007). “Video ergo sum: manipulating bodily self-consciousness”. *Science*, *317*(5841), 1096-1099.<br><br>(Lenggenhager et al., 2007)	(3) (4) It seemed as if I were feeling the touch of the highlighter in the location where I saw the virtual body/ mannequin/	(3) It seemed as if the touch I felt was caused by the highlighter touching the virtual body/ mannequin/ object.<br>Score 1 *	(1) It felt as if the virtual body/ mannequin/ object was my body.<br>Score 1 *	(4) It felt as if my (real) body was drifting towards the front (towards the virtual body/ mannequin/ object).<br>Score -1.5	(1) It seemed as if I might have had more than one body<br>Score -1.5	(3) (4) It seemed as if the touch I was feeling came from somewhere between my own body and the virtual body/ mannequin/	(4) It appeared (visually) as if the virtual body/ mannequin/ object were drifting backwards (towards the real body).			

	object touched.<br>Score 2.7 (from -3 to 3)<br>Significant difference between conditions *					object.<br>Score -2	Score -3			
Ehrsson, H. H. (2007). "The experimental induction of out-of-body experiences". *Science*, *317*(5841), 1048-1048.<br><br>(Ehrsson, 2007)	(5) The visual image of me started to change appearance so that I became (partly) transparent<br>Score -2.5 (from -3 to 3)<br>Significant difference from the middle score (4)*	(1) I could no longer feel my body, it was almost as if it had disappeared<br><br>Score -2 *	(3) (4) I did not feel the touch on my body but at some distance in space in front of me<br><br>Score -1.5 *	(4) I felt as if my head and body was at different locations, almost as if I had been "decapitated"<br><br>Score -2 *	(4) I experienced a movement sensation that I was floating from my real body to the location of the cameras<br><br>Score -2 *	(4) I experienced that my (felt) body was located at two locations at the same time<br><br>Score -0.5	(1) I felt I had two bodies<br><br>Score -1.5 *	(3) (4) I experienced that the hand I was seeing approaching the camera was directly touching my chest (with the rod)<br><br>Score 2.5 *	(1) (4) I felt as if my head and eyes were located at the same place as the cameras, and my body just below the cameras<br><br>Score 1.5 *	(4) I experienced that I was located at some distance behind the visual image of myself, almost as if I was looking at someone else<br><br>Score 1.5 *
Petkova, V. I., & Ehrsson, H. H. (2008). "If I were you: perceptual illusion of body swapping". *PloS one*, *3*(12), e3832.<br><br>(Petkova and Ehrsson, 2008)	(3) I seemed to feel the touch given to the mannequin<br>Score 1.5 (from -3 to 3)<br>Significant difference between conditions *	(3) It seemed as if the touch I felt was caused by the stick touching the mannequin's body<br>Score 2.5 *	(1) It felt like the mannequin's body was my body<br>Score 1.5 *	(5) I felt naked<br>Score -0.5 *	(1) I felt as if I had two bodies<br>Score -1 *	(5) I felt as if my body had turned into a plastic body<br>Score 0 *	(5) The mannequin's body began to resemble my own body in terms of shape, skin tone or some other visual feature.<br>Score -1			

Slater, M., et al. (2008). "Towards a digital body: the virtual arm illusion". *Frontiers in human neuroscience*, *2*, 6.<br><br>(Slater et al., 2008)	(3) (4) Sometimes I had the feeling that I was receiving the hits in the location of the virtual arm.<br>Score 6 (from 1 to 7) Significant difference between conditions *	(3) During the experiment there were moments in which it seemed as if what I was feeling was caused by the yellow ball that I was seeing on the screen.<br>Score 6*	(1) During the experiment there were moments in which I felt as if the virtual arm was my own arm.<br>Score 6*	(4) During the experiments there were moments in which it seemed that my real arm was being displaced towards the left (towards the virtual arm).<br>Score 1	(3) (4) During the experiment there were moments in which it seemed that the contact that I was feeling originated in some place in between my own arm and the virtual arm.<br>Score 3*	(1) (5) During the experiment there were moments in which I felt as if my real arm was becoming virtual.<br>Score 4*	(4) During the experiment there were moments in which it seemed (visually) that the virtual arm was being displaced towards the right (towards my real arm).<br>Score 3	(5) During the experiment there were moments in which the virtual arm started to look like my own arm in some aspects.<br>Score 5*	(1) During the experiment there were moments in which I had the sensation of having more than one right arm.<br>Score 1	
Fox, J., et al. (2009). "Virtual experiences, physical behaviors: The effect of presence on imitation of an eating avatar". *Presence : Teleoperators and Virtual Environments*, *18* (4), 294-303.<br><br>(Fox et al., 2009)	(1) To what extent do you feel the avatar is an extension of yourself?<br><br>Scores per question are not reported But there was aggregated significant difference between groups.	(6) To what extent do you feel that if something happens to the avatar, it feels like it is happening to you?	(1) To what extent do you feel you embodied the avatar?	(4) To what extent do you feel you were in the same room with the avatar?	(5) To what extent did the avatar seem real?					

Slater M, et al.(2010) “First Person Experience of Body Transfer in Virtual Reality”. PLoS ONE 5(5): e10564.<br><br>(Slater et al., 2010b)	(1) How much did you feel that the seated girl's body was your body?<br><br>Score 3 (from 1 to 5) Significant difference between conditions *	(3) How strong was the feeling that the woman you saw was directly touching your shoulder? Score 4*	(3) How strong was the feeling that the touch you felt was caused by the woman you saw? Score 3*	(5) How strong was the feeling that you were wearing different clothing, from when you started the experiment, while you were in the part of the room where the standing woman was located? Score 4*	(1) How strong was the feeling that the body of the girl in the mirror was your body? Score 3*	(1) When you were looking down from above how much did you feel a strong connection with the seated girl as if you were looking from at yourself? Score 2	(6) When the standing woman hit the seated woman, how much did you feel this as if this was an attack on your body? Score 2	(6) After you returned from looking down from above how much did you feel that the standing woman might hurt you? Score 4*		
González-Franco, M.,et al. (2010) ”The contribution of real-time mirror reflections of motor actions on virtual body ownership in an immersive virtual environment” .IEEE *VR,* (pp. 111-114).<br><br>(Gonzalez-Franco et al., 2010)	(1) I felt as if the body I saw in the mirror might be my body<br><br>Score 4.3 (from 1 to 5) Significant difference between conditions *	(6) I had the feeling that I might be harmed if I collided with the air fan<br><br>Score 2.6 *	(2) The movements I saw the body in the mirror make seemed to be my movements Score 4.5*	(1) The body I saw in the mirror was another person Score 2.8*	(1) (4) I felt as if my real body were floating above the ground. Score 3.9					

Lopez, C., et al. (2010). "How vestibular stimulation interacts with illusory hand ownership". *Con sciousness and cognition*, *19*(1), 33-47.<br><br>(Lopez et al., 2010)	(3) (4) It seemed as if I were feeling the touch of the paintbrush in the location where I saw the rubber hand touched<br><br>Score 5.2 (from 1 to 7) Significant difference between conditions *	(3) It seemed as if the touch I felt was caused by the paintbrush touching the rubber hand<br><br>Score 4.9*	(1) I felt as if the rubber hand were my hand<br><br>Score 4.6 *	(4) It felt as if my (real) hand were drifting towards the right (towards the rubber hand)<br><br>Score 2.7*	(1) It seemed as if I might have more than one left hand or arm<br><br>Score 2.4	(3) (4) It seemed as if the touch I was feeling came from somewhere between my own hand and the rubber hand<br><br>Score 2.5	(1) (5) It felt as if my (real) hand were turning 'rubbery<br><br>Score 3.4 *	(4) It appeared (visually) as if the rubber hand were drifting towards the left (towards my hand)<br><br>Score 1.9	(5) The rubber hand began to resemble my own (real) hand, in terms of shape, skin tone, freckles or some other visual feature<br><br>Score 4.1*	(1) I found myself liking the rubber hand<br><br>Score 4.1*
Petkova, V. I., et al. (2011). "The perspective matters! Multisensory integration in ego-centric reference frames determines full-body ownership ". *Frontiers in psychology*, *2*, 35.<br><br>(Petkova et al., 2011)	(3) I seemed to feel the touch given to the mannequin<br><br>Score 1.5 (from -3 to 3) Significant difference between conditions *	(3) It seemed as if the touch I felt was caused by the stick touching the mannequin's body<br><br>Score 2*	(1) It felt like the mannequin's body was my body<br><br>Score 1.5*	(5) I felt naked<br>Score 0	(1) I felt like I had two bodies (at the same time)<br>Score -2*	(5) It felt as if my body had turned into a plastic body<br>Score -2*	(1) (5) The mannequin's body began to resemble my own body in terms of shape, skin tone, or other visual features<br>Score 0*	(1) I felt like I was looking in a mirror<br>Score -2 *	(1) It felt as if the body I saw belonged to someone else<br>Score -1*	

van der Hoort, B., et al. (2011). “Being Barbie: the size of one’s own body determines the perceived size of the world”. *PloS one*, *6*(5), e20195.<br><br>(van der Hoort et al., 2011)	(1) I felt as if the artificial body was my body<br><br>Score 2 (from -3 to 3) Significant difference between conditions *	(3) It seemed as if the touch I felt was caused by the object touching the artificial body<br><br>Score 3 *	(3) It seemed as if I was feeling the touch that was applied to the artificial body<br>Score 2.5*	(1) I felt as if I had two bodies<br>Score -2.5*	(5) I felt younger than I actually am<br>Score -2	(5) I felt as if my body was turning artificial<br>Score -2	(5) The artificial body began to resemble my own body in terms of shape, skin tone or some other visual feature<br>Score -1	(6) The hand I saw appeared to me like a had of a giant<br>Score -3*	(6) The pencil appeared to be gigantic<br>Score -3*	
Normand, J. et al. (2011). “Multisensory stimulation can induce an illusion of larger belly size in immersive virtual reality.” *PloS one*, *6*(1), e16128.<br><br>(Normand et al., 2011)	(3) (4) It seemed as if I was feeling the touch at the location of the yellow ball.<br>Score 7 (from 1 to 7) Significant difference between conditions *	(3) It seemed as if the touch I felt was caused by the yellow ball touching the virtual body.<br>Score 7 *	(1) I felt as if the virtual body were my body.<br>Score 6*	(3) (4) It seemed as if the touch I was feeling was located somewhere between my felt body and the seen body<br>Score 4.5	(5) At some point during the experiment I felt my body expanding to take on the shape that I saw.<br>Score 6*	(5) I was aware of a conflict between my felt body and the seen body.<br>Score 3	(1) It seemed as if I had more than one body<br>Score 6	(5) After taking off the head-mounted display I felt the need to check that my body size was really smaller than the virtual body I had seen.<br>Score 3	(5) I felt an after-effect as if my body had become swollen.<br>Score 3	(5) The illusion of having a swollen body was very strong during the experience<br>Score 2
Kilteni, K., et al. (2012). “Extending body space in immersive virtual reality: a very long arm illusion”. PloS one, 7(7),	(3) (4) It seemed as if I were feeling the touch of the box in the location where I saw the virtual	(3) It seemed as if the touch I felt was from the box being touched by the virtual hand.	(1) I felt as if the virtual arm were my arm.<br>Score 4	(1) It felt as if I might have more than two arms.<br>Score 1	(3) (4) It seemed as if the touch I was feeling came from somewhere between my real and the virtual hand.	(5) It felt as if my real arm were becoming longer.<br>Score 2				

e40867.<br><br>(Kilteni et al., 2012b)	hand touching.<br><br>Score 5 (from 1 to 5) Significant difference between conditions *	Score 4*			Score 2					
Rosenberg, R. S., et al. (2013). "Virtual superheroes: Using superpowers in virtual reality to encourage prosocial behavior". *PloS one*, *8*(1), e55003.<br><br>(Rosenberg et al., 2013)	(3) I felt like I could reach out and touch the objects in the city<br><br>No significant difference reported.									
Pomes, A. and Slater, M., (2013). "Drift and ownership toward a distant virtual body". *Frontiers in human neuroscience*, *7*, p.908.<br><br>(Pomés and	(3) (4) It seemed as if I were feeling the touch of the ball in the location where I saw the virtual body touched	(3) It seemed as if the touch I felt was caused by the ball touching the virtual body<br><br>Score 4*	(1) It felt as if the virtual body was my body<br><br>Score 3	(4) It felt as if my (real) body was drifting toward the front (toward the virtual body)<br><br>Score 2	(1) It seemed as if I might have had more than one body<br><br>Score 2	(3) (4) It seemed as if the touch I was feeling came from somewhere between my own body and the virtual body<br><br>Score 2	(4) It appeared (visually) as if the virtual body was drifting backwards (towards the real body)<br><br>Score 2	(1) During the experiment the body I saw was that of another person<br><br>Score 3	(6) When the fan descended over the head of the virtual body in front, it felt as if it could chop my head<br><br>Score 3	

Slater, 2013)	Score 5 (from 1 to 5) Significant difference between conditions *									
Kilteni, K., et al (2013). “Drumming in immersive virtual reality: the body shapes the way we play”. *IEEE transactions on visualization and computer graphics*, *19*(4), pp.597-605.<br><br>(Kilteni et al., 2013)	(1) Even though the virtual body I saw did not look like me - I had the sensation that the virtual body I saw when I looked towards myself in the mirror was mine.	(1) Even though the virtual body I saw did not look like me - I had the sensation that the virtual body I saw when I looked down at myself was mine.	(1) (6) I had the sensation that I was with another person who was playing the drums.	(2) I felt myself to be more expressive than I normally am.	(2) I felt myself to be less expressive than I normally am.	(2) (5) My virtual body was more appropriate for playing this type of drumming than my real body.	(2) (5) My virtual body was less appropriate for playing this type of drumming than my real body.	(1) Even though the virtual body I saw did not look like me - overall I had the sensation that the virtual body I saw when I looked at myself in the mirror or when I looked down at myself was my body.		

Peck, T.C., et al. (2013). “Putting yourself in the skin of a black avatar reduces implicit racial bias”. *Consciousness and cognition*, *22*(3), pp.779-787.<br><br>(Peck et al., 2013)	(1) I felt as if the body I saw in the virtual world might be my body.	(6) I became nervous when the other avatars approached me.	(2) I felt like I controlled the avatar as if it was my own body.	(2) I liked being able to control the movements of the avatar.	(1) I felt like the avatar was not me.	(6) I wanted to say hello to the avatars as they walked past.				
Heydrich, L., et al (2013). “Visual capture and the experience of having two bodies–evidence from two different virtual reality techniques”. *Frontiers in psychology*, *4*, 946.<br><br>(Heydrich et al., 2013)	(3) (4) It seemed as if I were feeling the touch of the stick in the location where I saw the two virtual bodies touched<br><br>Score 7 (from 1 to 7) Significant difference between conditions *	(3) It seemed as if the touch I felt was caused by the stick touching the two virtual bodies<br><br>Score 6*	(1) I felt as if the two virtual bodies where my body<br><br>Score 6*	(4) I felt as if my (real) body was drifting forwards (towards the two virtual bodies)<br><br>Score 2	(1) It seemed as if I might have more than one body<br><br>Score 3*	(3) (4) It seemed as if the touch I was feeling came from somewhere between my own body and the two virtual bodies<br><br>Score 3	(4) It appeared (visually) as if the two virtual bodies were drifting backwards (towards my body)<br>Score 2	(1) Did you identify with 1. the right virtual body more. 2. the left virtual body more. 3. both virtual bodies	(1) Did you identify with the two avatars 1. at the same time, 2. at different times	

Kokkinara, E. and Slater, M. (2014). “Measuring the effects through time of the influence of visuomotor and visuotactile synchronous stimulation on a virtual body ownership illusion”. *Perception*, *43*(1), pp.43-58.<br><br>(Kokkinara and Slater, 2014)	(4) Overall, I felt as if my legs were located where I saw the virtual legs to be.<br><br>Score 7 (from 1 to 7) Significant difference between conditions *	(1) Overall, I felt that the virtual legs were my own legs.<br><br>Score 7 *	(2) The movements of the virtual legs were caused by my movements.<br><br>Score 7 *	(3) It seemed as if the touch I felt was caused by the red ball touching my body.<br><br>Score 7 *	(6) I was stressed when I saw the table being pulled away.<br><br>Score 4	(6) (1) I felt like my own legs were affected when I saw the table being pulled away.<br><br>Score 4	(1) It seemed as if I might have more than two legs.<br><br>Score 1	(1) Overall, I felt that the virtual legs belonged to someone else.<br><br>Score 1 *		
Banakou, D., & Slater, M. (2014). “Body ownership causes illusory self-attribution of speaking and influences subsequent real speaking.” *Proceedings of the National Academy of Sciences*, *111*(49), 17678-17683.<br><br>(Banakou and Slater, 2014)	(1) I felt that the virtual body I saw when looking down at myself was my own body<br>Score 2 (from -3 to 3) Significant difference between conditions *	(1) I felt that the virtual body I saw when looking at myself in the mirror was my own body<br>Score 2 *	(5) I felt that my virtual body resembled my own (real) body in terms of shape, skin tone, or other visual features<br>Score 0	(1) I felt as if I had two bodies<br><br>Score -2	(2) I felt that the movements of the virtual body were caused by my own movements<br><br>Score 3*	(2) It felt as if the voice I heard was coming from somewhere in the room<br><br>Score -1.5 *	(2) It felt as if the voice I heard was coming from inside my head<br><br>Score 1*	(2) It felt as if the voice I heard was my own voice<br><br>Score 1 *	(2) It felt as if the voice I heard was a modified version of my own voice<br><br>Score 2	(2) It felt as if I was speaking out the words I heard<br><br>Score 3*

Maselli, A. and Slater, M., (2014). "Sliding perspectives: dissociating ownership from self-location during full body illusions in virtual reality". *Frontiers in human neuroscience*, *8*, p.693.<br><br>(Maselli and Slater, 2014)	(1) I felt that the body I saw was my own body<br><br>Score 6 (from 1 to 7) Significant difference between conditions *	(5) I felt that I was wearing different clothing than when I came to the laboratory<br><br>Score	(1) I felt as if the body I saw belonged to someone else<br><br>Score 3*	(1) I felt as if I had two bodies	(4) During the experiment, I felt as if my real body was drifting to the left<br><br>Score 1*	(4) During the experiment, I felt as if my real body was drifting to the right	(4) It seemed as if I was at two places at the same time	(6) (2) When I was the virtual legs separating I felt as though my real legs were moving<br><br>Score 4 *	(6) I felt a weird sensation in my legs when I saw the virtual legs separating<br><br>Score 5*	(6) (2) When I saw the virtual legs spreading apart, I felt as the instinct to spread apart my real legs<br><br>Score 6*
	(3) It seemed as though the touch I felt was caused by the carton tube that I was seeing moving on the virtual body	(3) (4) It seemed as though I felt the touch in the location where the carton tube touched the virtual legs<br><br>Score 7*								
Piryankova, I.V., et al (2014). "Owning an overweight or underweight body: distinguishing the physical, experienced and	(1) I felt as if the virtual body was my body<br><br>Scores per question are not reported But there	(1) I experienced the virtual body as my body	(1) I had the feeling that I was looking at myself	(5) During the experiment I felt heavier than usual	(1) I experienced the arms of the virtual body as parts of myself	(1) I experienced the legs of the virtual body as parts of myself	(1) I had the feeling that I had a strong connection with the virtual body	(5) I was not aware that my physical body was different than the virtual body	(1) It felt as if I had more than one body	(1) I felt myself somehow connected to the virtual body

virtual body”. *PloS one*, *9*(8), p.e103428.<br><br>(Piryankova et al., 2014)	was aggregated significant difference between groups.									
	(1) I experienced the virtual body as myself	(5) It felt like my physical body was changing to take on the shape of the virtual body	(5) During the experiment I felt my physical body had become bigger	(1) I had the feeling that I and the virtual body where the same	(3) (4) I had the sensation as if I was feeling the touch at the location at which the left virtual leg was stroked	(3) (4) I had the sensation as if I was feeling the touch at the location at which the right virtual leg was stroked	(3) (4) It felt like I was feeling touch at the same time as the virtual body was touched	(3) (4) It felt as if the touch I was feeling was located somewhere between my physical body and my virtual body location	(6) (3) I had the feeling that the arm I was seeing was directly touching me	(3) (4) I had the sensation as if I was feeling the touch at the location at which the right virtual arm was stroked
	(3) (6) I had the sensation as though the touch I felt was caused by the arm touching the virtual body	(3) (6) I had the feeling that the touch I felt was caused by the arm I saw	(4) I had the feeling that I was sitting in the same location as the virtual body	(1) (4) I felt as if I was inside the virtual body	(2) I felt I could move the virtual body if I wanted to	(2) I had the feeling that I had control over the virtual body				

González-Franco, M., et al. (2014). "A threat to a virtual hand elicits motor cortex activation." *Experimental brain research*, *232*(3), pp.875-887.<br><br>(González-Franco et al., 2014)	(1) I felt as if the hand I saw in the virtual world might be my hand.<br><br>Score 5 (from 1 to 5) Significant difference from the middle score (3)*	(6) I had the feeling that I might be harmed when I saw the knife inside the hand.<br>Score 1*	(6) I had the feeling that I might be harmed when I saw the knife outside the hand.<br>Score 3	(1) The hand I saw was the hand of another person.<br>Score 2*	(6) I saw the knife as a threat to my body.<br><br>Score 3					
Falconer, C. J., et al. (2014). "Embodying compassion: a virtual reality paradigm for overcoming excessive self-criticism." *PloS one*, *9*(11), e111933.<br><br>(Falconer et al., 2014)	(1) I felt as if the body I saw when I looked down was my own body<br><br>Score 1 (from -3 to 3) Significant difference between conditions *	(1) I felt as if the body I saw when I looked in the mirror was my own body<br><br>Score 1*	(1) I had the feeling that I was looking at myself in the mirror rather than looking at someone else<br><br>Score 0*	(1) How much did you feel like you had two bodies?<br><br>Score -1	(2) The movements of the virtual body responded to the movements of my real body.<br><br>Score 2 *					

Osimo, S. A., et al (2015). “Conversations between self and self as Sigmund Freud—A virtual body ownership paradigm for self counselling”. *Scientific reports*, *5*, 13899<br><br>(Osimo et al., 2015)	(1) (5) Even though the body I see might not physically look like me, I feel that the virtual body I see when I look down towards myself is my body.<br><br>Score 6 (from 1 to 7) Significant difference between conditions *	(1) (5) Even though the body I see might not physically look like me, I feel that the virtual body I see reflected in the mirror is my body.<br><br>Score 6 *	(2) I feel that the movements of the virtual body are caused by my own movements.<br><br>Score 7*	(5) The body I see in the virtual world physically looks like me.<br><br>Score 6*						
Kokkinara, E., et al. (2016). “First Person Perspective of Seated Participants Over a Walking Virtual Body Leads to Illusory Agency Over the Walking.” *Scientific Reports*, *6*.<br><br>(Kokkinara et al., 2016)	(4) During the experiment I felt as if my body was located where I saw the virtual body to be.<br><br>Score 6 (from 1 to 7) Significant difference between	(1) During the experiment I felt that the virtual body was my own body.<br><br>Score 6*	(4) (5) During the experiment I felt that I was standing upright.<br><br>Score 6	(2) During the experiment I felt that the leg movements of the virtual body were my movements. Score 5*	(2) During the experiment I felt that the leg movements of the virtual body were caused by my movements.<br><br>Score 5*	(1) During the experiment I felt that the virtual body belonged to someone else.<br><br>Score 2.5*	(2) I felt that I was moving through space rather than the world moving past me.<br><br>Score 6	(2) I felt that I was walking.<br><br>Score 6	(2) I felt that I was being dragged. Score 2	(2) I felt that I was sliding

	conditions *									
Padrao, G., Gonzalez-Franco, M., et al. (2016). "Violating body movement semantics: Neural signatures of self-generated and external-generated errors." *NeuroIm age*, *124*, 147-156.<br><br>(Padrao et al., 2016)	(1) It felt as if the virtual body was my body<br><br>Score 2 (from -3 to 3) Significant difference between conditions.	(4) I felt as if my hand was located where I saw the virtual hand to be<br><br>Score 2.5*	(1) It seemed as if I might have had more than one body<br><br>Score -1	(4) It seemed as if the position of the hand I was feeling came from somewhere between my own hand and the virtual hand<br><br>Score -1	(2) Most of the time, the movements of the virtual hand seemed to be my movements<br><br>Score 2*	(2) Sometimes, I felt that the movements of the virtual hand were influencing my own movements<br>Score -2	(2) Sometimes, the virtual hand seemed to be moving by itself<br>Score -2*	(5) It sometimes felt as if my real hand was turning 'virtual'<br><br>Score 0	(2) (6) It seems sometimes that the errors were not caused by myself<br><br>Score -2*	
Banakou, D., et al. (2016). "Virtual Embodiment of White People in a Black Virtual Body Leads to a Sustained Reduction in Their Implicit Racial Bias". *Frontiers in Human Neuroscience*, *10*<br><br>(Banakou et al., 2016)	(1) I felt that the virtual body I saw when looking down at myself was my own body<br><br>Score 1.5 (from -3 to 3) Significant difference between conditions*.	(1) I felt as if I had two bodies<br><br>Score -2	(1) I felt that the virtual body I saw when looking at myself in the mirror was my own body<br><br>Score 2*	(5) I felt that my virtual body resembled my own (real) body in terms of shape, skin tone or other visual features<br><br>Score -3*	(2) I felt that the movements of the virtual body were caused by my own movements					

Maselli, A., et al. (2016). “The sense of body ownership relaxes temporal constraints for multisensory integration.” *Scientific Reports*, *6*.<br><br>(Maselli et al., 2016)	(1) I felt as if the virtual hand I was looking at was my own hand<br><br>Score 2 (from -3 to 3) Significant difference between conditions*	(3) I felt as if my finger was touching the virtual wheel<br><br>Score 1.5*	(3) I felt as if the rotation of the virtual wheel produced the tactile stimuli on my finger<br><br>Score 1	(2) I felt as if the virtual arms were following the movements of my own arms<br><br>Score 3*	(1) I felt as if I had two right hands<br><br><u>Score -3</u>					
Argelaguet, F., et al (2016). “The role of interaction in virtual embodiment: Effects of the virtual hand representation.” In *Virtual Reality (VR), 2016 IEEE* (pp. 3-10). IEEE.<br><br>(Argelaguet et al., 2016)	(2) I felt as if the virtual representation of the hand moved just like I wanted it to, as if it was obeying my will.<br>Score 5.7 (from 1 to 7) Significant difference between conditions*	(2) I expected the virtual representation of the hand to react in the same way as my own hand<br><br>Score 6.09*	(2) I felt like I was able to interact with the environment the way I wanted to.<br><br>Score 5.79*	(2) I felt that the interaction with the environment was realistic<br><br>Score 5.1.	(2) I felt like I controlled the virtual representation of the hand as if it was part of my own body.<br><br>Score 4.8	(1) I felt as if the virtual representation of the hand was part of my body<br><br>Score 5.5*	(1) I felt as if the virtual representation of the hand was someone else’s<br><br>Score 2.2	(2) I felt that I was losing the control of my hand when the virtual hand was not responding properly	(6) I thought that the virtual representation of the hand could be harmed by the virtual danger	(6) I felt that my real body was endangered during the experiment.

	(6) Did you try to avoid the virtual obstacle while performing the task?<br><br>Score 5.9	(6) I felt my own hand tickling whenever the virtual representati on of the hand went through a virtual obstacle.<br><br>Score 2.1	(2) I felt that the virtual representati on of the hand was able to go through the virtual obstacles<br><br>Score 4.3.							
Bourdin, P., et al. (2017). “A Virtual Out-of-Body Experience Reduces Fear of Death.” *PloS one*, *12*(1), e0169343.<br><br>(Bourdin et al., 2017)	(1) I felt as if the body I was seeing was my own body<br><br>Score 4 (from 1 to 7) Significant difference between conditions*	(1) I felt as if the body I was seeing belonged to someone else<br><br>Score 4.5 *	(4) I felt as if I was floating in air	(4) I felt as if I was in an elevated position in the room	(1) I felt a connection with the body, as if I was looking down at myself.<br><br>Score 5 *	(1) (4) I felt as if I had an invisible body	(4) I felt out of my body<br><br>Score 6 *			
