# Supplementary material for: Avatar Embodiment. Towards a Standardized Questionnaire
Source: Front Robot AI. 2018 Jun 22;5:74. doi: 10.3389/frobt.2018.00074 (PMC7805666; doi:10.3389/frobt.2018.00074)
Supplement: Supplementary file 2 [file Data_Sheet_2.PDF]

Appendix 2. Embodiment Questionnaire

Participant ID ______________ Condition _____________

Please select your level of agreement with the following statements:

"During the experiment there were moments in which…

Q1. I felt as if the virtual body I saw when I looked down was my body"

strongly disagree	*disagree*	*somewhat disagree*	*neither agree nor disagree*	*somewhat agree*	agree	strongly agree
(-3)	*(-2)*	*(-1)*	*(0)*	(1)	(2)	(3)

Q2. It felt as if the virtual body I saw was someone else"

strongly disagree	*disagree*	*somewhat disagree*	*neither agree nor disagree*	*somewhat agree*	agree	strongly agree
(-3)	*(-2)*	*(-1)*	*(0)*	(1)	(2)	(3)

Q3. It seemed as if I might have more than one body"

strongly disagree	*disagree*	*somewhat disagree*	*neither agree nor disagree*	*somewhat agree*	agree	strongly agree
(-3)	*(-2)*	*(-1)*	*(0)*	(1)	(2)	(3)

Q4. I felt as if the virtual body I saw when looking in the mirror was my own body"

strongly disagree	*disagree*	*somewhat disagree*	*neither agree nor disagree*	*somewhat agree*	agree	strongly agree
(-3)	*(-2)*	*(-1)*	*(0)*	(1)	(2)	(3)

Q5. I felt as if the virtual body I saw when looking at myself in the mirror was another person"

strongly disagree	*disagree*	*somewhat disagree*	*neither agree nor disagree*	*somewhat agree*	agree	strongly agree
(-3)	*(-2)*	*(-1)*	*(0)*	(1)	(2)	(3)

Q6. It felt like I could control the virtual body as if it was my own body"

strongly disagree	*disagree*	*somewhat disagree*	*neither agree nor disagree*	*somewhat agree*	agree	strongly agree
(-3)	*(-2)*	*(-1)*	*(0)*	(1)	(2)	(3)

Q7. The movements of the virtual body were caused by my movements"

strongly disagree	*disagree*	*somewhat disagree*	*neither agree nor disagree*	*somewhat agree*	agree	strongly agree
(-3)	*(-2)*	*(-1)*	*(0)*	(1)	(2)	(3)

Q8. I felt as if the movements of the virtual body were influencing my own movements"

strongly disagree	*disagree*	*somewhat disagree*	*neither agree nor disagree*	*somewhat agree*	agree	strongly agree
(-3)	*(-2)*	*(-1)*	*(0)*	(1)	(2)	(3)

Q9. I felt as if the virtual body was moving by itself"

strongly disagree	*disagree*	*somewhat disagree*	*neither agree nor disagree*	*somewhat agree*	agree	strongly agree
(-3)	*(-2)*	*(-1)*	*(0)*	(1)	(2)	(3)

Q10. It seemed as if I felt the touch of the ____ in the location where I saw the virtual body touched"

strongly disagree	*disagree*	*somewhat disagree*	*neither agree nor disagree*	*somewhat agree*	agree	strongly agree
(-3)	*(-2)*	*(-1)*	*(0)*	(1)	(2)	(3)

Q11. It seemed as if the touch I felt was located somewhere between my physical body and the virtual body"

strongly disagree	*disagree*	*somewhat disagree*	*neither agree nor disagree*	*somewhat agree*	agree	strongly agree
(-3)	*(-2)*	*(-1)*	*(0)*	(1)	(2)	(3)

Q12. It seemed as if the touch I felt was caused by the ____ touching the virtual body"

strongly disagree	*disagree*	*somewhat disagree*	*neither agree nor disagree*	*somewhat agree*	agree	strongly agree
(-3)	*(-2)*	*(-1)*	*(0)*	(1)	(2)	(3)

Q13. It seemed as if my body was touching the ____"

strongly disagree	*disagree*	*somewhat disagree*	*neither agree nor disagree*	*somewhat agree*	agree	strongly agree
(-3)	*(-2)*	*(-1)*	*(0)*	(1)	(2)	(3)

Q14. I felt as if my body was located where I saw the virtual body"

strongly disagree	*disagree*	*somewhat disagree*	*neither agree nor disagree*	*somewhat agree*	agree	strongly agree
(-3)	*(-2)*	*(-1)*	*(0)*	(1)	(2)	(3)

Q15. I felt out of my body"

strongly disagree	*disagree*	*somewhat disagree*	*neither agree nor disagree*	*somewhat agree*	agree	strongly agree
(-3)	*(-2)*	*(-1)*	*(0)*	(1)	(2)	(3)

Q16. I felt as if my (real) body were drifting towards the virtual body or as if the virtual body were drifting towards my (real) body"

strongly disagree	*disagree*	*somewhat disagree*	*neither agree nor disagree*	*somewhat agree*	agree	strongly agree
(-3)	*(-2)*	*(-1)*	*(0)*	(1)	(2)	(3)

Q17. It felt as if my (real) body were turning into an 'avatar' body"

strongly disagree	*disagree*	*somewhat disagree*	*neither agree nor disagree*	*somewhat agree*	agree	strongly agree
(-3)	*(-2)*	*(-1)*	*(0)*	(1)	(2)	(3)

Q18. At some point it felt as if my real body was starting to take on the posture or shape of the virtual body that I saw"

strongly disagree	*disagree*	*somewhat disagree*	*neither agree nor disagree*	*somewhat agree*	agree	strongly agree
(-3)	*(-2)*	*(-1)*	*(0)*	(1)	(2)	(3)

Q19. At some point it felt that the virtual body resembled my own (real) body, in terms of shape, skin tone or other visual features".

strongly disagree	*disagree*	*somewhat disagree*	*neither agree nor disagree*	*somewhat agree*	agree	strongly agree
(-3)	*(-2)*	*(-1)*	*(0)*	(1)	(2)	(3)

Q20. I felt like I was wearing different clothes from when I came to the laboratory"

strongly disagree	*disagree*	*somewhat disagree*	*neither agree nor disagree*	*somewhat agree*	agree	strongly agree
(-3)	*(-2)*	*(-1)*	*(0)*	(1)	(2)	(3)

Q21. "I felt that my own body could be affected by ____"

strongly disagree	*disagree*	*somewhat disagree*	*neither agree nor disagree*	*somewhat agree*	agree	strongly agree
(-3)	*(-2)*	*(-1)*	*(0)*	(1)	(2)	(3)

Q22. I felt a ____sensation in my body when I saw ____"

strongly disagree	*disagree*	*somewhat disagree*	*neither agree nor disagree*	*somewhat agree*	agree	strongly agree
(-3)	*(-2)*	*(-1)*	*(0)*	(1)	(2)	(3)

Q23. When ____ happened, I felt the instinct to ____"

strongly disagree	*disagree*	*somewhat disagree*	*neither agree nor disagree*	*somewhat agree*	agree	strongly agree
(-3)	*(-2)*	*(-1)*	*(0)*	(1)	(2)	(3)

Q24. I felt as if my body had ____"

strongly disagree	*disagree*	*somewhat disagree*	*neither agree nor disagree*	*somewhat agree*	agree	strongly agree
(-3)	*(-2)*	*(-1)*	*(0)*	(1)	(2)	(3)

Q25. I had the feeling that I might be harmed by the ____ "

strongly disagree	*disagree*	*somewhat disagree*	*neither agree nor disagree*	*somewhat agree*	agree	strongly agree
(-3)	*(-2)*	*(-1)*	*(0)*	(1)	(2)	(3)
